# Psychological impact of genetic eligibility for CFTR modulator therapy in children with cystic fibrosis and their parents 

**DOI:** 10.1007/s00431-026-06929-z

**Published:** 2026-04-15

**Authors:** Berrak Oztosun, Gizem Durcan, Simay Buse Gulumser, Defne Altug, Basak Gunal, Elif Anac, Mesut Yavuz, Haluk Cokugras, Burak Dogangun, Ayşe Ayzit Kilinc Sakalli

**Affiliations:** 1 Department of Pediatric Pulmonology, Cerrahpasa Medical Faculty, Istanbul University-Cerrahpasa, Istanbul, Turkey; 2 Department of Pediatrics, Cerrahpasa Medical Faculty, Istanbul University-Cerrahpasa, Istanbul, Turkey; 3 Department of Child and Adolescent Psychiatry, Cerrahpasa Medical Faculty, Istanbul University-Cerrahpasa, Istanbul, Turkey

**Keywords:** Cystic fibrosis, CFTR modulators, Modulator eligibility, Psychological burden

## Abstract

Cystic fibrosis (CF) is a genetic disorder with significant physical and psychological impacts. CFTR modulators (CFTRm) have transformed CF care. Although CFTRm have transformed CF care, genetic eligibility creates distinct patient experiences. This study evaluates the psychological impact of genetic CFTRm eligibility status on children with CF and their caregivers. This cross-sectional study included 118 children with CF and their primary caregivers. Psychological assessments were performed by the child psychiatry team. Children completed the Child Attitude Toward Illness Scale (CATIS), Brief Illness Perception Questionnaire (BIPQ-Patient), and Children’s Hope Scale (CHS). Caregivers completed the Beck Hopelessness Scale (BHS), Strengths and Difficulties Questionnaire (SDQ), and BIPQ-Caregiver. Clinical and demographic data were collected, and analyses were stratified according to CFTRm genetic eligibility and the presence of bronchiectasis. Among participants, 53.39% (*n* = 63) were genetically eligible for CFTRm, while only 35.59% (*n* = 42) were actively receiving treatment. Genetically eligible children demonstrated higher levels of hope and a more positive perception of their illness compared with genetically ineligible children. Among patients without bronchiectasis, psychological outcomes did not differ between eligibility groups. In contrast, among those with bronchiectasis, modulator-eligible children reported greater hope and more positive attitudes toward their disease, and their mothers also reported higher levels of hope.

*Conclusion*: Genetic eligibility for CFTRm is associated with meaningful differences in psychological well-being among children with CF, particularly in those with more advanced lung disease. These findings suggest that eligibility status itself may function as a psychosocial determinant and should be considered when delivering patient-centered care for children with CF and their families.

**Table:** 

**What is Known:** • *CF is associated with increased psychological burden in both children and their caregivers.* • *Although CFTR modulators have transformed cystic fibrosis care, access remains limited, and real-world use may be affected by factors such as genetic eligibility, treatment adherence, and potential adverse effects.*
**What is New:** • *Children with CF who are genetically eligible for CFTR modulator therapy—particularly those with advanced lung disease—report higher levels of hope, more positive attitudes toward their illness, and lower perceived illness threat than ineligible peers.*

**What is Known:**

• *CF is associated with increased psychological burden in both children and their caregivers.*

• *Although CFTR modulators have transformed cystic fibrosis care, access remains limited, and real-world use may be affected by factors such as genetic eligibility, treatment adherence, and potential adverse effects.*

**What is New:**

• *Children with CF who are genetically eligible for CFTR modulator therapy—particularly those with advanced lung disease—report higher levels of hope, more positive attitudes toward their illness, and lower perceived illness threat than ineligible peers.*

## Introduction

Cystic fibrosis (CF), one of the most common autosomal recessive diseases among Caucasians, results from mutations in the cystic fibrosis transmembrane conductance regulator (CFTR) gene. CF results from structural and functional abnormalities in the CFTR protein, an epithelial ion channel responsible for chloride and bicarbonate transport. Consequently, this leads to multisystem impairment and a shortened life expectancy [1]. Chronic airway infection and inflammation can lead to progressive structural lung damage, most notably bronchiectasis, impairing mucociliary clearance, declining lung function, increasing respiratory symptoms, and reducing quality of life [2].

The International Depression Epidemiological Study (TIDES), which screened over 6000 patients and 4000 parents, found that depression and anxiety occur 2–3 times more frequently in people with cystic fibrosis (pwCF) and their parents than in the general population. Psychological distress in pwCF has been linked to lower treatment adherence, worse CF-related health outcomes, and reduced quality of life. Consequently, the Cystic Fibrosis Foundation and the European Cystic Fibrosis Society created guidelines for screening and managing depression and anxiety in patients and caregivers [3].

More recently, CFTR modulators (CFTRm), designed to enhance CFTR protein function, have revolutionized care for pwCF and ushered in a new era of CF management [1]. However, despite these advances, a substantial number of individuals with CF worldwide remain untreated. Non-use of CFTRm therapy may result from several factors, including genetic ineligibility, limited access due to insurance and financial barriers despite genetic eligibility, medical contraindications preventing treatment initiation, or discontinuation due to adverse effects, including hepatic, dermatologic, and neuropsychiatric reactions. Particularly in low-income countries, the lack of reimbursement policies and the high cost of CFTRm significantly limit access to treatment [4]. While approximately 89% of eligible patients in the USA are reported to have access to CFTRm therapy, global access remains markedly limited, with estimates suggesting that only around 12% of the worldwide CF population receives these treatments [5]. This inequality poses a substantial global challenge when viewed through the lens of ethical and human rights principles and has profound physical and psychological consequences for patients. The inability to access treatment can lead to stress, anxiety, and feelings of hopelessness among patients.

The aim of this study was to examine the psychological impact of genetic eligibility for CFTRm therapy on children with CF and their parents. By focusing on differences between genetically eligible and ineligible patients, we aimed to explore how eligibility itself may influence illness perceptions, emotional and behavioral outcomes, and disease-related beliefs in patients and their families.

## Methods

### Participants

The study included children and adolescents diagnosed with CF who were followed at the Istanbul University-Cerrahpaşa, Cerrahpaşa Faculty of Medicine (IUC CTF) Pediatric Pulmonology Outpatient Clinic, along with their parents. Throughout the duration of the study from January to September 2024, all 230 families were contacted and invited to participate. Of these, 118 families agreed to participate and were included in the study. Reasons for non-participation among the remaining 112 families included declining participation (*n* = 32) and inability to attend the study visit within the data collection period (*n* = 80). A total of 118 mothers and 118 fathers were approached; due to the death of two mothers and two fathers, 116 of each were included.

Of the 118 participating children, 66 were aged ≥ 8 years and completed the self-report measures, while 116 mothers and 116 fathers completed caregiver questionnaires.

Of the 63 patients identified as genetically eligible for CFTRm therapy, 42 were on therapy at the time of data collection, and 21 were not. During the study period, CFTRm were not covered by the national state-funded health insurance system in Türkiye. Access required individual legal application with associated legal and administrative costs. Consequently, genetic eligibility did not necessarily correspond to treatment use.

### Study procedure

Our clinic’s child psychiatry team administered validated psychiatric scales face-to-face to ensure consistency and reliability in the evaluation process. In line with the study’s focus on genetic eligibility status, instruments assessing hope, hopelessness, illness perception, and behavioral and emotional difficulties were selected, as these domains were considered relevant to the potential psychological implications of modulator eligibility.

All participants’ mothers and fathers completed the Strengths and Difficulties Questionnaire (SDQ) to assess children’s psychological problems, the caregiver version of the Brief Illness Perception Questionnaire (BIPQ-Caregiver) to evaluate their perceptions of the child’s illness, and the Beck Hopelessness Scale (BHS) to assess their own hopelessness. Children over 8 years of age were asked to complete the Child Attitude Toward Illness Scale (CATIS), the Brief Illness Perception Questionnaire for Patients (BIPQ-Patient), and the Children’s Hope Scale (CHS).

In addition to psychiatric scales, we assessed demographic and clinical data recorded in our patient files, including pulmonary function test results, body mass index (BMI), pseudomonas colonization and bronchiectasis status, history of hospitalization in the past year, diabetes status, genetic eligibility for CFTRm, and use of CFTRm.

Patients were divided into two subgroups based on their eligibility for the CFTRm, as assessed using the Vertex Treatment Finder, which provides variant-specific information on responsiveness to currently approved CFTRm. Comparisons of psychological outcomes were performed by genetic eligibility status.

Bronchiectasis was identified based on radiologist-confirmed findings on thoracic high-resolution computed tomography (HRCT). Because bronchiectasis reflects greater disease severity, subgroup analyses were performed accordingly.

The protocol was approved by Istanbul University-Cerrahpasa, Cerrahpasa Medical Faculty Ethics Committee (Decision No: 1078897 dated 02.09.2024). Parents and their children were informed about the scales and procedures. Written informed consent was obtained from parents, and age-appropriate written assent was obtained from children aged 8 years and older.

### Psychological measures

#### Strengths and difficulties questionnaire

The SDQ, developed by Goodman in 1997, is a 25-item scale to assess children and adolescents’ behavioral and emotional difficulties [6]. It consists of five subscales: Hyperactivity/Inattention, Conduct Problems, Emotional Symptoms, Peer Problems, and Prosocial Behavior. The first four subscales contribute to the Total Difficulties Score, with a total score range of 0–40 (each subscale ranges 0–10). While high scores on the prosocial behavior subscale indicate a positive individual strength, high scores on the first four subscales indicate severe individual difficulties. Güvenir et al. [7] conducted the Turkish validity and reliability study, and Cronbach’s alpha values ranged from 0.37 to 0.84 [7].

#### Child attitude toward illness scale

CATIS was developed by Austin and Huberty in 1993, which measures children’s attitudes toward having a chronic illness [8]. It consists of 13 items and is rated on a 5-point Likert scale, yielding a total score range of 13–65, with higher scores reflecting a more positive perception of illness. The Turkish validity and reliability study was conducted by Ersun in 2010, and Cronbach’s alpha value is 0.79 [9].

#### Brief Illness Perception Questionnaire (BIPQ)

BIPQ was originally developed by Weinman and colleagues in 1996 and later revised by Moss-Morris et al. in 2002. It is a 9-item scale designed to assess an individual’s perception of their illness [10, 11]. Each item is scored from 0 to 10, yielding a total score of 0–90, with higher scores indicating a more negative perception of illness. A validity and reliability study has been conducted in Turkish, and the Cronbach’s alpha value is 0.94 [12]. In this study, both self-report and parent-report forms were used.

#### Beck hopelessness scale

The BHS, developed by Beck et al. [13], evaluates pessimism and expectations about the future [13]. It consists of 20 true/false statements, producing a total score range of 0–20, with higher scores indicating greater hopelessness. Seber et al. [14] validated the Turkish adaptation, and Cronbach’s alpha value is 0.86 [14].

#### Children’s hope scale

The CHS was developed by Snyder et al. in 1997, which assesses the levels of hopefulness in children [15]. The scale includes six items scored on a 1–6 scale, producing a total score range of 6–36, with higher totals indicating stronger levels of hope. The Turkish version of the scale was validated by Atik and colleagues in 2009, and Cronbach’s alpha value is 0.74 [16].

### Statistical analysis

All statistical analyses were performed using IBM SPSS Statistics (IBM Corp., Armonk, NY, USA). Descriptive statistics were used to summarize demographic and clinical characteristics. Continuous variables are presented as median (interquartile range) or mean ± standard deviation, as appropriate. The Shapiro–Wilk test was used to assess normality. For group comparisons, independent-samples *t*-tests or Mann–Whitney *U* tests were used for continuous variables, and chi-square tests were used for categorical variables. One-way ANOVA or Kruskal–Wallis tests were conducted for multi-group comparisons, followed by post hoc analysis (Tukey or Dunn-Bonferroni) when appropriate. A *p*-value of less than 0.05 was considered statistically significant. Because bronchiectasis represents greater structural disease severity in CF, a prespecified subgroup analysis was planned to examine whether disease severity modified the association between CFTRm eligibility and psychological outcomes.

## Results

### Participant characteristics

Sixty-three (53.39%) patients were genetically eligible for CFTRm, but only 42 (35.59%) were actively using the therapy. Despite being eligible for CFTRm, 21 patients (17.79%) were not receiving the treatment. For all 21 patients, non-use of CFTRm was attributable to financial barriers.

Among the 118 participants, 44.9% were male. The median age of the participants was 113 months (69.75–179). The median age at diagnosis was 3 months (1–12), while the median follow-up duration was 95 months (63.75–162.5). The clinical characteristics of the patients are summarized in Table 1.

**Table 1:** Summary of demographic and characteristics of study participants

**Variable**	Median (IQR 25–75) or mean (SD) or *n* (%)
Age (months)	113 (69.75–179)
Sex, male	53 (44.9%)
Age at diagnosis (months)	3 (1–12)
Follow-up duration (months)	95 (63.75–162.5)
CFTRm usage—*n* (%)	42 (35.6%)
IVA—*n* (%)	3 (2.5%)
TEZ/IVA—*n* (%)	1 (0.8%)
ELX/TEZ/IVA—*n* (%)	38 (32%)
BMI-SDS	− 0.34 (1.37)
PFT compatible	65 (55.1%)
FEV1 (%)	82.72% (24.75)
FVC (%)	87.2% (20.66)
FEV1/FVC (%)	94.07% (12.77)
Pseudomonas infection—*n* (%)	70 (59.3%)
Pseudomonas colonization—*n* (%)	29 (24.6%)
Pancreas insufficiency—*n* (%)	109 (92.4%)
CF-related diabetes—*n* (%)	9 (7.6%)
Hospitalization over the past year	
None—*n* (%)	82 (69.5%)
1—*n* (%)	31 (26.3%)
≥ 2—*n* (%)	5 (4.2%)
Bronchiectasis	
Yes (%)	41 (34.7%)
No (%)	77 (65.3%)

*BMI*, body mass index; *CF*, cystic fibrosis; *CFTRm*, cystic fibrosis transmembrane conductance regulator modulator; *ELX*, elexacaftor; *FEV*_*1*_, forced expiratory volume in 1 s; *FVC*, forced vital capacity; *IVA*, ivacaftor; *LUM*, lumacaftor; *PFT*, pulmonary function test; *TEZ*, tezacaftor

### Comparison of patients according to CFTR modulator eligibility

Demographic and clinical characteristics by modulator eligibility are shown in Table 2, with no significant differences between groups (*p* > 0.05 for all).

**Table 2:** Demographic and clinical characteristics of study participants by CFTR modulator eligibility

Variable	Modulator eligible (*n* = 63)	Modulator ineligible (*n* = 55)	*t*/*z*	*p*
	Median (IQR 25–75) or mean (± SD) or *n* (%)			
Patient age (months)	122 (70–208)	109 (69–175)	− 0.642	0.521^a^
Age at diagnosis (months)	3 (1–12)	3 (1–11)	− 0.393	0.695^a^
Follow-up duration (months)	97 (62–171)	94 (65–149)	− 0.129	0.897^a^
Sex, male	27 (%42.85)	26 (%47.27)		0.630^c^
BMI-SDS	− 0.14 (± 1.33)	− 0.58 (± 1.40)	1.713	0.90^b^
FEV1 (%)	82.80 (± 24.29) (*n* = 36)	82.62 (± 25.74) (*n* = 29)	0.30	0.976^b^
FVC (%)	87 (± 22.26) (*n* = 36)	87.44 (± 18.86) (*n* = 29)	− 0.086	0.932^b^
FEV1/FVC (%)	95.27 (± 11.61) (*n* = 36)	92.58 (± 14.14) (*n* = 29)	0.843	0.403^b^
Pseudomonas infection	42 (66.66%)	28 (50.90%)		0.082^c^
Pseudomonas colonization	15 (23.80%)	14(27.27%)		0.836^c^
Pancreas insufficiency	60 (87.30%)	49 (89.09%)		0.209^c^
CF-related diabetes	5(7.93%)	4 (7.27%)		0.892^c^
Hospitalization over the past year	0 (0–1)	0 (0–1)		0.276^c^
Bronchiectasis	23 (36.5%)	18 (32.72%)		0.667
**Mother** Age (years)	*n* = 63 39.34 (± 7.30)	*n* = 53 38.33(± 7.24)	0.735	0.464^b^
Formal education				
Below high school—*n* (%)	35 (55.55%)	43 (78.18)		**0.010**^c^
High school and above—*n* (%)	28 (44.44%)	12 (30%)		
**Father** Age (years)	*n* = 63 42.98 (± 7.68)	*n* = 53 42.39 (± 8.14)	0.396	0.693^b^
Formal education				
Below high school—*n* (%)	31 (49.20%)	35 (63.63%)		0.115^c^
High school and above—*n* (%)	32 (50.79%)	20 (36.36%)		

^a^Mann-Whitney *U* test; ^b^independent-samples *T* test; ^c^chi-square test

*BMI*, body mass index; *CF*, cystic fibrosis; *FEV*_*1*_, forced expiratory volume in 1 s; *FVC*, forced vital capacity

Bold values indicate statistically significant results (*p *< 0.05)

#### Positive psychological outcomes

Positive psychological outcomes, CHS, and CATIS were first analyzed according to CFTR modulator eligibility.

The CHS score was significantly higher in the eligible group compared to the ineligible group (*t* = 2.644, *p* = 0.010) (Table 3).

**Table 3:** Psychological scale scores of patients and parent-reported child outcomes according to CFTR modulator eligibility

Variable	Modulator eligible *n* = 63	Modulator ıneligible *n* = 55	*t*/*z*	*p*
	Mean ± SD or median (IQR 25–75)			
Patient-report	*N* = 37	*N* = 29		
CHS	27.13 (± 5.6)	22.86 (± 7.52)	2.644	**0.010**^b^
CATIS	3.51 (± 0.65)	3.36 (± 0.64)	0.953	0.344^b^
BIPQ	32.63 (± 11.44)	40.10 (± 15.11)	− 2.265	**0.027**^b^
Mother-report	*N* = 63	*N* = 53		
SDQ	10 (7–13)	13 (9 − 17)	− 2.649	**0.008**^a^
SDQ subscales				
Emotional symptoms	2 (1–4)	3 (1 − 5)	− 1.591	0.112^a^
Conduct problems	1 (0–2)	2 (1 − 3)	− 1.696	0.090^a^
Hyperactivity/inattention	3 (2–5)	4 (3 − 6)	− 1.697	0.090^a^
Peer relationship problems	2 (1–4)	3 (2 − 5)	0.076	0.076^a^
Prosocial behavior	9 (7–10)	8 (6 − 9)	0.158	0.158^a^
Father-report	*N* = 63	*N* = 53		
SDQ	9 (6.5–13)	10 (6 − 15)	− 0.269	0.788^a^
SDQ subscales				
Emotional symptoms	2 (1–3.5)	2 (1 − 5)	0.345	0.345^a^
Conduct problems	1 (0–2)	1 (0 − 2)	0.720	0.720^a^
Hyperactivity/inattention	3 (2–5)	4 (2 − 6)	0.380	0.380^a^
Peer relationship problems	3 (2–4)	2 (1 − 3)	0.091	0.091^a^
Prosocial behavior	8 (6–9)	8 (7 − 10)	0.115	0.115^a^
Mother-self-report				
BHS	3 (2–5)	4 (2 − 6)	− 0.462	0.644^a^
BIPQ	40 (34–47)	41 (35 − 47.25)	− 0.624	0.533^a^
Father-self-report				
BHS	3 (2–6)	3 (2 − 6)	− 0.407	0.684^a^
BIPQ	41 (31.5–48)	43 (35.75 − 50)	− 1.297	0.195^a^

^a^Mann-Whitney *U* test; ^b^independent-samples *T* test

*BHS*, Beck Hopelessness Scale; *BIPQ*, Brief Illness Perception Questionnaire; *CATIS*, Child Attitude Toward Illness Scale; *CHS*, Children’s Hope Scale; *SDQ*, Strengths and Difficulties Questionnaire

Bold values indicate statistically significant results (*p *< 0.05)

When patients were stratified according to bronchiectasis status, those in the bronchiectasis group who were eligible for CFTRm had higher CHS (*z* =  − 2.689, *p* = 0.007) and CATIS scores (*z* =  − 2.603, *p* = 0.009). Among patients without bronchiectasis, no significant differences were observed between eligible and non-eligible groups in CHS or CATIS scores (*p* > 0.05) (Table 4).

**Table 4:** Psychological scale scores of patients and parent-reported child outcomes according to CFTR modulator eligibility and bronchiectasis status

	Bronchiectasis +				Bronchiectasis −			
Variable	Modulator eligible	Modulator non-eligible	***t*/*z***	*p*	Modulator eligible	Modulator non-eligible	*t/z*	*p*
	Mean ± SD or median (IQR 25–75)				Mean ± SD or median (IQR 25–75)			
Patient-report	*N* = 21	*N* = 15			*N* = 16	*N* = 14		
CHS	26 (22–32.5)	20 (13–27)	− 2.689	**0.007**	28 (25–31.75)	27 (24.5–31)	− 0.334	0.739
CATIS	3.54 (2.84–4.15)	2.92 (2.38–3.15)	− 2.603	**0.009**			− 0.604	0.546
BIPQ	35 (26.5–42.75)	42 (37–54)	− 2.569	**0.010**	32 (24–43.5)	31.5 (23–41.25)	− 0.291	0.771
Mother-report	*N* = 23	*N* = 18			*N* = 40	*N* = 35		
SDQ	8.5 (7–11.25)	16 (10.75–21)	− 2.945	**0.003**	10 (7–13)	11 (8.5–15)	− 1.089	0.276
SDQ subscales								
Emotional symptoms	2 (1–4)	4 (2–6)	− 2.343	**0.019**	2.5 (1–4)	2 (1–4)	− 0.377	0.706
Conduct problems	1 (0–3)	3 (0.75–4.25)	− 1.764	0.078	1 (0–2)	2 (0.5–3)	− 0.794	0.427
Hyperactivity/inattention	2.5 (2–5.25)	4 (3–7)	− 2.440	0.15	3.5 (2–5)	4 (2.5–5)	− 0.330	0.741
Peer relationship problems	2 (1.75–4)	3 (2–5.25)	− 1.776	0.076	2 (1–3.75)	3 (1–4.5)	− 0.936	0.349
Prosocial behavior	8.5 (6.75–9.25)	8.5 (4–10)	− 0.042	0.967	9 (8–10)	8 (6–9)	− 1.823	0.068
Father-report	*N* = 23	*N* = 18			*N* = 40	*N* = 35		
SDQ	10 (8.25–16.75)	9 (2.8–11)	− 0.754	0.451	9 (6–11)	9 (6–12.75)	− 0.039	0.969
SDQ subscales								
Emotional symptoms	2.5 (1.25–4.5)	2 (1–5)	− 0.152	0.879	1 (0.5–3.5)	2 (1–4.75)	− 1.201	0.230
Conduct problems	1 (0–2)	1 (1–2)	− 1.260	0.208	2 (1–2.5)	2 (1–3)	− 1.310	0.190
Hyperactivity/inattention	4 (2.25–5.75)	4 (3–6)	− 0.186	0.853	3 (2–4)	3.5 (2–5)	− 1.101	0.271
Peer relationship problems	3 (2–4)	3 (1–5)	− 0.236	0.987	3 (2–4)	2 (1–3)	− 1.754	0.080
Mother-self-report	*N* = 23	*N* = 18			*N* = 40	*N* = 37		
BHS	4 (2–5)	5.5 (3.75–10)	− 2.209	**0.027**	3 (2–5)	3 (1.5–5)	− 0.955	− 0.057
BIPQ	44 (38–47)	43 (40–49)	− 0.699	0.485	38.5 (31–45)	40 (34.25–46.75)	− 0.516	0.606
Father-self-report								
BHS	5 (2–9)	6 (2–9)	− 0.587	0.557	9 (6–11)	9 (6–12.75)	− 0.326	0.744
BIPQ	45.5 (35.25–48.75)	48 (42–56)	− 1.703	0.089	41 (29.5–47.5)	41 (32–50)	− 0.654	0.513

^a^Mann-Whitney *U* test; ^b^independent-samples *T* test

*CHS*, Children’s Hope Scale; *CATIS*, Child Attitude Toward Illness Scale; *BIPQ*, Brief Illness Perception Questionnaire; *SDQ*, Strengths and Difficulties Questionnaire; *BHS*, Beck Hopelessness Scale; *BIPQ*, Brief Illness Perception Questionnaire

Bold values indicate statistically significant results (*p* < 0.05)

#### Negative psychological outcomes

Negative psychological outcomes, BIPQ, SDQ, and BHS, were then evaluated.

The BIPQ-patient score was significantly higher in the ineligible group (*t* =  − 2.265, *p* = 0.027).

In the mother-report, SDQ total scores were significantly higher in genetically ineligible patients (*z* =  − 2.649, *p* = 0.008). However, no significant differences were observed in SDQ subscale scores (emotional symptoms, conduct problems, hyperactivity/inattention, peer relationship problems, prosocial behavior) between the groups (*p* > 0.05 for all) (Table 3).

In the bronchiectasis subgroup, BIPQ scores were significantly higher in non-eligible patients compared to eligible patients (*z* =  − 2.569, *p* = 0.010). In the bronchiectasis group, mothers of non-eligible children had significantly higher BHS scores (*z* =  − 2.209, *p* = 0.027) and higher total SDQ scores (*z* =  − 2.945, *p* = 0.003). SDQ subscale analysis revealed that emotional symptoms were significantly higher in the non-eligible group (*z* =  − 2.343, *p* = 0.019).

Among patients without bronchiectasis, no significant differences were observed between eligible and non-eligible groups in either patient- or parent-reported measures (*p* > 0.05) (Table 4).

No significant difference was found in the father-report scale scores (Table 3).

## Discussion

In this single-center cohort of children with CF and their families, genetic eligibility for CFTRm therapy was associated with higher levels of hope and more positive illness perception. Notably, this association was not observed uniformly across all participants; instead, it was most evident among children with bronchiectasis. In parallel with these child-reported findings, mothers of genetically ineligible children—especially those with bronchiectasis—reported greater emotional and behavioral difficulties and more pronounced feelings of hopelessness. In this study, children who were genetically eligible for CFTRm demonstrated significantly higher CHS scores than those who were ineligible, indicating greater hope. The difference in CHS scores between modulator-eligible and ineligible children was particularly evident among patients with bronchiectasis. In this subgroup, modulator-eligible children also had higher CATIS scores, suggesting that CFTRm eligibility may be associated with a more positive illness attitude. The perception that a treatment option is available can itself boost hope by providing a potential pathway toward improved disease management. In addition, expectations regarding treatment effectiveness are an important component of illness perception and may shape how children interpret and respond to their illness [17, 18]. Today, hope and optimism are recognized not only as positive emotions but also as significant factors in physical and mental health. Previous studies have shown that hope and positive illness attitudes are associated with better psychological adjustment and improved quality of life in children with chronic diseases [19, 20].

Patients, ineligible for CFTRm, had higher BIPQ-Patient scores, indicating a more negative illness perception. Patients’ subjective understanding of their illness, known as illness perception, plays a critical role in psychological adaptation and overall well-being. Illness perception significantly influences their coping strategies and health outcomes. Viewing sickness as manageable and well-understood is associated with better psychological well-being; conversely, seeing it as threatening and unpredictable is linked to emotional distress and ineffective coping. In a study conducted by Sawicki et al. (2010) [21], illness perceptions were found to be strongly associated with psychosocial aspects of health-related quality of life (HRQOL) in adults with CF, but not with physical aspects. Specifically, patients who perceived their illness as comprehensively understood and manageable reported higher emotional and social well-being, regardless of their objective clinical status [22].

Mothers of children ineligible for CFTRm reported higher SDQ scores, indicating greater emotional and behavioral difficulties in their children. However, it appears that modulator eligibility does not impact fathers’ SDQ scores. Previous studies generally show good agreement between maternal and paternal reports; however, some research indicates that mothers and fathers report different levels of difficulty. As noted in the meta-analysis by Achenbach et al., despite overall consistency between maternal and paternal reports, factors such as the disorder’s clinical presentation, cultural background, and variations in family structure can lead to differences in perspective between parents. This difference may also be explained by the fact that mothers are often more directly involved in the daily care of children with chronic illnesses, which may allow them to observe psychosocial difficulties more closely and sensitively than fathers. Therefore, it is important to evaluate both maternal and paternal perspectives to obtain more accurate and detailed information [23, 24].

In the bronchiectasis subgroup, BIPQ scores were significantly higher in non-eligible patients compared to eligible patients. Additionally, mothers of patients with bronchiectasis who are ineligible for CFTRm report higher SDQ scores, indicating greater emotional and behavioral difficulties in their children. Notably, mothers of patients with bronchiectasis who are ineligible for CFTRm have higher BHS scores, potentially indicating more hopelessness when their child is genetically ineligible for these therapies. In a qualitative study by von Scheven et al. [25], patients with chronic disease and their parents described hope as an evolving and adaptable process. They mentioned that external factors, such as access to treatment, care coordination, and supportive communication with healthcare providers, were important to their sense of hope. For many families, the prospect of a life-changing therapy or the presence of a caring clinician acted as a vital source of renewed hope, particularly in cases of progressive or advanced disease [26, 27].

On the contrary, among patients without bronchiectasis, no significant differences were found in any of the scales between eligible and non-eligible groups, suggesting that the psychological relevance of modulator eligibility may be more pronounced in patients with an irreversible and considerable complication such as bronchiectasis. This emphasizes that the severity of the disease not only has a direct psychological impact but may also amplify the effects of other stressors through a synergistic interaction. In other words, patients experiencing more severe forms of the illness may be more vulnerable to additional challenges—such as ineligibility to a life-changing treatment—leading to greater differences in psychological outcomes.

The psychological consequences of modulator ineligibility have also been highlighted in previous studies. Kramer-Golinkoff et al. reported that individuals who were not eligible for CFTRm frequently experienced emotional distress and feelings of marginalization while observing peers benefit from treatments that remained inaccessible to them. Similarly, recent research has shown that patients who were ineligible for or unable to access CFTRm had significantly higher rates of depression and anxiety compared with those who had access to these therapies [28, 29].

These findings should also be interpreted within the broader context of global inequities in access to CFTRm therapies. Although more than half of our cohort was genetically eligible for CFTRm, only one-third were receiving treatment, and all untreated eligible patients were unable to access therapy because of financial barriers. This reflects a wider global disparity, with substantially lower CFTRm use reported in several Eastern European and middle-income countries, including Türkiye, compared with Western Europe [30]. In addition, some patients with rare CFTR variants may remain untreated because these mutations are not included in current FDA- or EMA-approved indications, despite emerging evidence suggesting potential responsiveness to modulator therapy [31, 32]. A small proportion of genetically eligible patients may also remain untreated because of treatment intolerance or adverse effects. Taken together, these factors suggest that both health-system barriers and biological variability may influence real-world access to CFTR modulators and may help explain some of the differences observed in psychological outcomes among children with CF and their families.

This study has several limitations that need to be considered. The cross-sectional design of the research has limited ability to assess causality between modulator eligibility and psychosocial outcomes. We were unable to assess how these measures may fluctuate over time, particularly in relation to acute exacerbations or disease progression. Longitudinal studies are required to better demonstrate the effects of treatment access on psychological well-being. The cross-sectional quantitative design does not allow for a detailed exploration of the underlying reasons for subgroup differences. Qualitative studies may provide deeper insight into the psychosocial mechanisms underlying these findings. Furthermore, although standardized and validated scales were used, self-reported measures may be affected by reporting biases. Finally, our analysis focused on a single cohort at a CF center in Türkiye, and the results may not be directly applicable to populations with different cultural or health care contexts.

CFTRm has been a groundbreaking discovery in the treatment of CF, changing the global perspective on the disease. Numerous aspects have progressed for patients who once spent much of their time in hospitals. Many of them are now living lives that feel far more normal. Along with the physical improvements, simply knowing they might live longer and have more independent lives seems to make a significant emotional difference. For many pwCF, these therapies represent more than just medical treatment—they offer something harder to measure—a sense of hope for the future [33, 34].

## Conclusion

This study demonstrates that children with CF who are genetically eligible for CFTRm therapy—especially those with bronchiectasis—report higher hope levels, more positive attitudes toward their illness, and lower perceived illness threat compared with ineligible peers. Mothers of eligible patients also report lower levels of psychological difficulties in their children. In patients without bronchiectasis, however, genetic eligibility was not associated with significant differences in psychological measures, suggesting that the emotional relevance of eligibility is more pronounced in the context of advanced disease.

Advances in CF care have reshaped expectations for the future, with CFTRm representing a significant milestone in disease management. Genetic ineligibility for treatment does not just mean missing out on it; it also shapes how patients and their families perceive the illness, how they cope, and how much hope they have for the future. From this perspective, eligibility can be viewed as a psychosocial determinant that shapes emotional adjustment, particularly among patients with more advanced disease, and warrants consideration within patient-centered models of care.

## Data Availability

No datasets were generated or analysed during the current study.
